# Case Report: Developmental-like skeletal deformities and transient osteosclerosis as rare presentations of primary hyperparathyroidism

**DOI:** 10.3389/fendo.2026.1672914

**Published:** 2026-02-13

**Authors:** Peijun Deng, Qin Wang, Miaoxian Huang, Haiying Wu, Jianwen Huang, Jie Li, Xiaosheng Gao, Mi Zhou, Yunfa Yang, Junbo Liang

**Affiliations:** 1 Department of Orthopaedic Surgery, Guangzhou First People’s Hospital, the Second Affiliated Hospital, South China University of Technology, Guangzhou, China; 2 Department of Respiratory and Critical Care, Mianyang People’s Hospital, Mianyang, China; 3 Department of Hematology, Tongde Hospital of Zhejiang Province, Hangzhou, China

**Keywords:** osteotomy, parathyroidectomy, primary hyperparathyroidism, skeletal deformities, transient osteosclerosis

## Abstract

**Background:**

Skeletal disorders in primary hyperparathyroidism (PHPT) classically manifest with osteoporosis or brown tumors. While skeletal deformities are documented in resource-limited areas, coxa vara combined with genu valgum remains unreported.

**Case Presentation:**

A 14-year-old Tibetan female from rural China presented with progressive bilateral coxa vara (left: 102.45°, right: 109.10°) and genu valgus (left: 24.20°, right: 18.80°). Staged osteotomies for lower limb deformities were planned. After the stage 1 osteotomy, severe hypercalcemia (peak: 3.79 mmol/L; reference: 2.11–2.52 mmol/L) and elevated PTH (995 pg/mL; reference: 15–65 pg/mL) emerged, leading to Single-photon emission computed tomography (SPECT) confirmed diagnosis of a left inferior parathyroid adenoma. Following the multidisciplinary team (MDT) recommendation, parathyroidectomy (PTX) achieved a biochemical cure, though postoperative hypocalcemia (nadir: 1.62 mmol/L; reference: 2.11–2.52 mmol/L) required aggressive calcium and calcitriol supplementation. After metabolic stabilization, the stage 2 osteotomy was performed. Notably, multifocal osteosclerotic regions around the knees emerged during the X-ray follow-up, resolving within 1 year with rigorous perioperative calcium and vitamin D management. One-year follow-up confirmed complete bony union, functional recovery, and deformity correction.

**Conclusion:**

This case highlights two key points: first, PHPT can masquerade as developmental skeletal deformity in adolescents, mandating a high index of suspicion and biochemical screening. Second, we describe a novel, self-resolving phenomenon of transient postoperative osteosclerosis, likely representing a reparative bone response following biochemical cure. Successful orthopedic correction before full metabolic normalization underscores the feasibility of staged management with meticulous perioperative care. These observations expand the phenotypic spectrum of pediatric PHPT.

## Introduction

Primary hyperparathyroidism (PHPT) is a rare endocrine disorder of excessive parathyroid hormone (PTH) secretion that results in hypercalcemia and its associated renal and skeletal complications (1, 2). The clinical profile of PHPT has evolved significantly since the 1970s due to widespread biochemical screening (3). However, delayed diagnosis persists in resource-limited areas, often presenting with skeletal deformities, pathologic fractures, or renal impairment (4, 5). Although​ the 2022 American Society for Bone and Mineral Research (ASBMR) guidelines significantly refined​ the understanding of PHPT epidemiology, pathophysiology, and management (6), critical gaps persist, particularly in managing severe skeletal deformities. For instance, severe coxa vara combined with genu valgum remains undocumented (7, 8). Furthermore, while parathyroidectomy (PTX) remains definitive therapy (6), postoperative skeletal changes are conventionally confined​ to hypocalcemic states seen in hungry bone syndrome (9, 10) —leaving other bone responses unexplored.

Here, we report a diagnostically challenging case of PHPT in a 14-year-old Tibetan female from rural China. Uniquely, her presentation featured: (1) Progressive bilateral coxa vara and genu valgus initially misdiagnosed as developmental deformity; (2) Transient multifocal osteosclerosis emerging post-parathyroidectomy—a radiographic finding never described in PHPT literature. This case expands the phenotypic spectrum of PHPT and proposes a novel bone-reparative response following biochemical cure.

## Case description

### Patient information

A 14-year-old Tibetan female from rural Tibet, China, presented with a 10-year history of progressive bilateral lower limb deformities and gait disturbance. Born with normal limb morphology and function, she developed bilateral coxa vara and genu valgum during childhood, culminating in ambulatory dysfunction and squatting difficulty. The patient reported no history of trauma, fractures, renal disease, or endocrine disorders, and there was no family history of genetic conditions.

### Clinical findings

**Figure 1:**
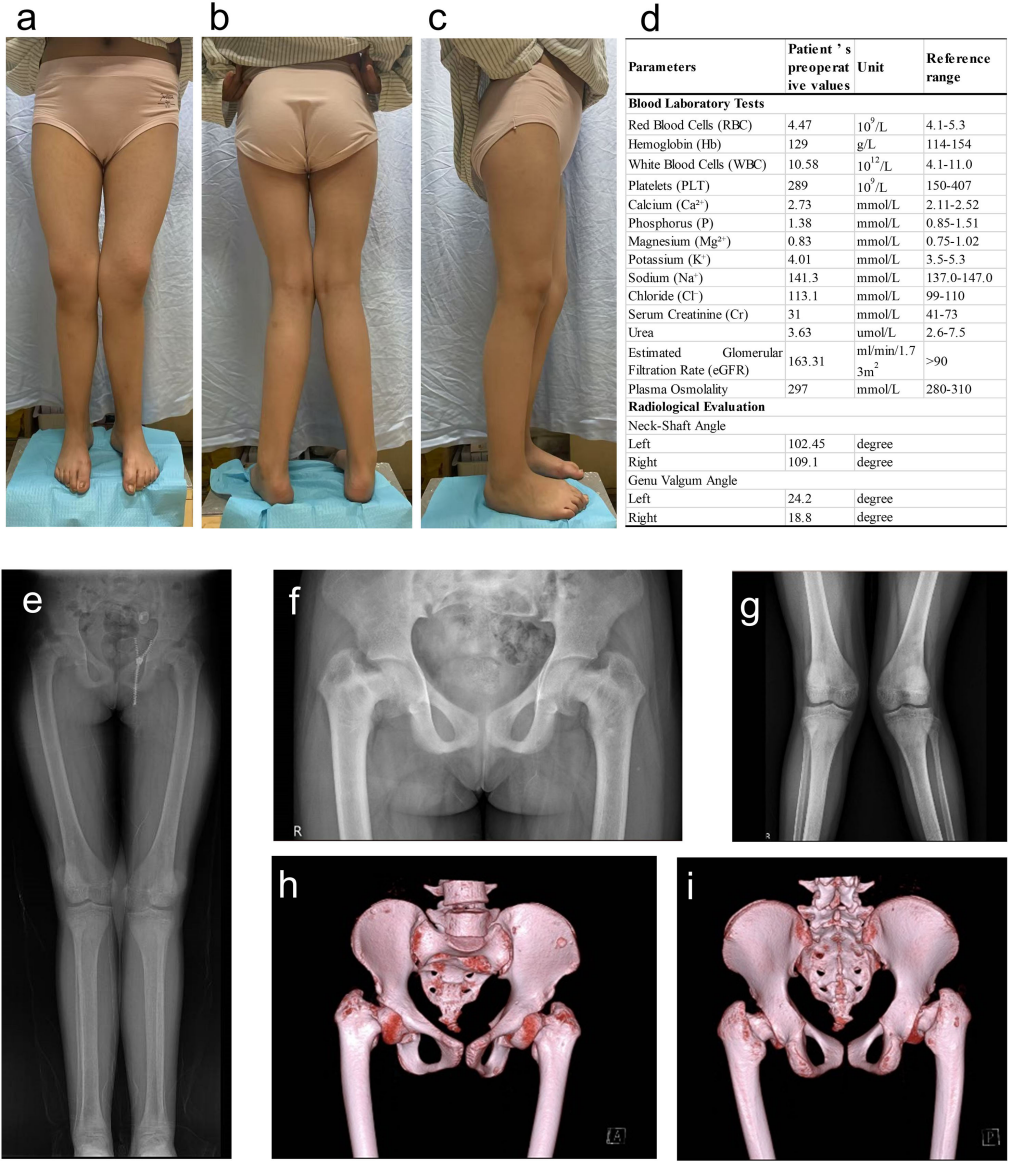
Clinical Findings at admission. **(a–c)** Clinical photographs (anterior, posterior, and lateral views) showing bilateral coxa vara and genu valgus. **(d)** Laboratory tests and imaging results; **(e)** Full-length lower limb radiograph. **(f, g)** Anteroposterior (AP) radiographs of the hip and knee joints. **(h, i)** 3D CT reconstruction of the hip joints.

On presentation, she (BMI: 26.75) exhibited bilateral coxa vara and genu valgum (**Figures 1a–c**), abnormal gait, without joint pain or neurological deficits. Laboratory tests at admission showed slightly elevated serum calcium (2.73 mmol/L, reference: 2.11–2.52 mmol/L), normal phosphorus (1.38 mmol/L, reference: 0.85–1.51 mmol/L), as well as normal albumin, creatinine, urea, and estimated glomerular filtration rate (eGFR) (**Figure 1d**). X-ray and CT scan (**Figures 1e–i**) confirmed severe skeletal deformities without evidence of tumors, infections, or pathological fractures. Developmental skeletal deformity was initially diagnosed, and staged corrective osteotomies were planned. Stage 1: Bilateral proximal femoral lateral closing-wedge osteotomies below the femoral trochanter with plate fixation. Stage 2 (performed after systemic stabilization and postoperative knee valgus reassessment): Angle-adjusted supracondylar femoral osteotomies with plate fixation.

### Medical interventions

#### Stage 1 osteotomy

**Figure 2:**
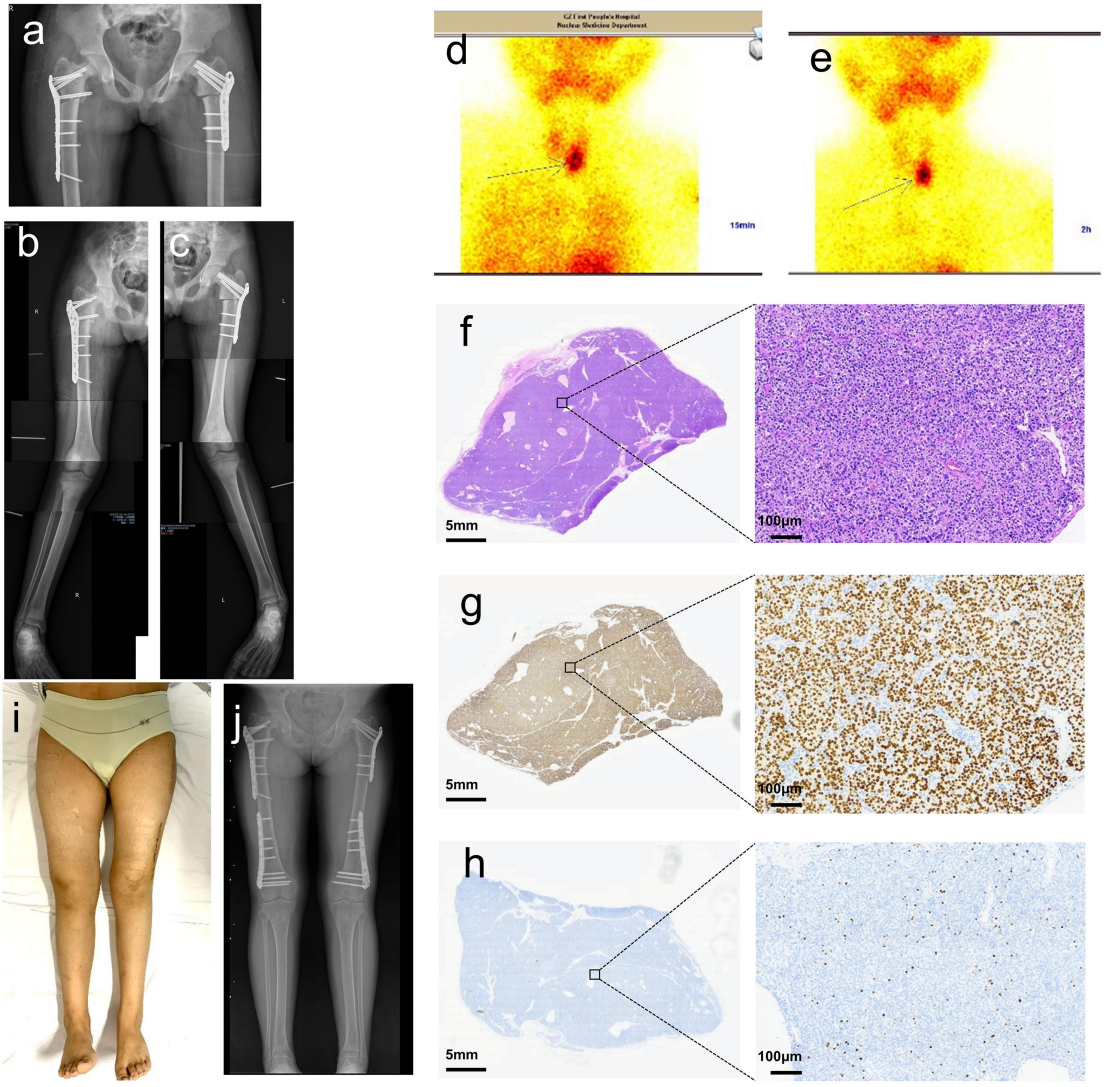
Postoperative findings after osteotomies and parathyroidectomy (PTX). **(a–c)** Postoperative radiographs after stage 1 osteotomy, demonstrating corrected coxa vara and residual genu valgus. **(d, e)** Single-photon emission computed tomography (SPECT) confirmed a left inferior parathyroid adenoma. **(f)** Histopathology: H&E staining shows nodular parathyroid hyperplasia. **(g)** Immunohistochemical staining shows strong nuclear positivity for GATA-3, confirming the parathyroid origin of the cells. **(h)** Immunohistochemical staining shows a Ki-67 proliferation index of approximately 10%, which is relatively high for a typical parathyroid adenoma and may reflect a more active proliferative state in this young patient with hyperplastic histology. **(i)** Clinical photograph showing restored lower limb alignment after two-stage correction. **(j)** Radiographs at the 1-year follow-up demonstrated complete bony union and maintained alignment.

Bilateral proximal femoral lateral closing-wedge osteotomies were performed (left: 25°, right: 18°) with plate and screw fixation (**Figures 2a–c**). Postoperatively, on day 5, the patient developed severe hypercalcemia (3.79 mmol/L; reference: 2.11–2.52 mmol/L) and hypophosphatemia (0.58 mmol/L; reference: 1.45–2.10 mmol/L), unresponsive to daily intravenous furosemide (20 mg). Given the patient’s young age, concerns regarding potential nephrotoxicity of bisphosphonates in the immediate postoperative setting with fluctuating renal perfusion, and the lack of immediate availability of calcimimetics (e.g., cinacalcet) in our hospital formulary, intramuscular salmon calcitonin (50 IU/day) was initiated as a rapid-acting bridging therapy to lower serum calcium while urgent preparation for parathyroidectomy was underway. This choice was based on calcitonin’s known rapid onset of action in inhibiting osteoclastic bone resorption, providing a temporary measure ahead of definitive surgery. Further evaluation revealed markedly elevated PTH (995 pg/mL; reference: 15–65 pg/mL), with normal renal function (creatinine: 56 μmol/L; reference: 44-97 μmol/L, urea: 4.2 mmol/L; reference: 2.8-7.2 mmol/L, eGFR: 98 mL/min/1.73 m²; reference: 90–120 mL/min/1.73m²). 99mTc-MIBI Single-photon emission computed tomography (SPECT) identified a 2.6×1.8 cm left inferior parathyroid adenoma (**Figures 2d, e**). A multidisciplinary team confirmed PHPT, and PTX was recommended.

#### PTX

On the 15th day after stage 1 osteotomy, the left inferior parathyroid adenoma was resected, and frozen section confirmed the excised tissue as parathyroid. Intraoperative PTH levels decreased from 995 pg/mL to 51.5 pg/mL (reference: 15–65 pg/mL) within 10 minutes, confirming surgical success. Histopathology confirmed a benign parathyroid adenoma (**Figures 2f–h**). Post-PTX, the patient developed severe hypocalcemia, hypomagnesemia, and hypophosphatemia, requiring aggressive supplementation with intravenous calcium gluconate and magnesium sulfate, as well as oral calcitriol (**Table 1**).

**Table 1:** Clinical timeline of diagnosis and management​.

​Date​	​Medical event​	​Key findings/Interventions​	​Clinical decision basis​
​2009​-03-21	Birth	Normal limb morphology and function	Maternal/pediatric records
​2014​	Symptom onset (age 5)	Progressive bilateral lower limb deformities	Parental recall; no prior medical evaluation
​2024-01-01​	Initial hospital admission	• Coxa vara (L:102.45°, R:109.1°) • Genu valgus (L:24.2°, R:18.8°) • Serum Ca^2+^: 2.73 mmol/L (reference: 2.11–2.52 mmol/L)	Developmental deformity suspected
​2024-01-10​	Stage 1 osteotomy	Bilateral proximal femoral osteotomies (L:25°, R:18°)	Pre-op planning for deformity correction
​2024-01-15​	Post-osteotomy day 5	• ​Severe hypercalcemia (Ca^2+^: 3.79 mmol/L; reference: 2.11–2.52 mmol/L)​ • PTH 995 pg/mL (reference: 15–65 pg/mL) • IV Furosemide 20 mg/day + IM Calcitonin 50 IU/day started	Unresponsive to hydration
​2024-01-25​	Parathyroidectomy (PTX)	• Left inferior parathyroid adenoma resected • Intra-op PTH 51.5 pg/mL (reference: 15–65 pg/mL)	SPECT confirmation + MDT consensus
​2024-01-27​	Post-PTX day 2	• Hypocalcemia (Ca^2+^:1.62 mmol/L; reference: 2.11–2.52 mmol/L) • Hypomagnesemia (Mg^2+^:0.52 mmol/L; reference: 0.7–1.1 mmol/L) • IV Ca Gluconate 12 g/day + IV MgSO_4_ 15 g/day + Oral Calcitriol 0.5 µg/day	Hungry bone syndrome prophylaxis
​2024-02-01​	Stage 2 osteotomy	Bilateral distal femoral osteotomies (L:26°, R:18°)	Metabolic stabilization (Ca^2+^ >2.0 mmol/L; reference: 2.11–2.52 mmol/L)
​2024-02-12​	Post-osteotomy week 2	• Ca^2+^: 2.15 mmol/L; reference: 2.11–2.52 mmol/L • IV calcium gluconate reduced to 6 g/day • IV MgSO_4_ discontinued	Normalizing serum electrolytes
​2024-02-27​	Follow-up X-ray (post-PTX day 33)	Multifocal osteosclerotic lesions around the knees	Routine imaging surveillance
​2024-03-01​	Outpatient management	• Ca^2+^: 2.28 mmol/L; reference: 2.11–2.52 mmol/L • Oral CaCO_3_ discontinued • Calcitriol 0.25 µg/day	Stable bone metabolism
​2024-07​	6-month follow-up	Independent ambulation achieved	Physical therapy assessment
​2025-01​	1-year follow-up	• Complete resolution of osteosclerosis • Bony union confirmed • Hardware removed	Final radiographic/functional evaluation

#### Stage 2 osteotomy

The timing of the stage 2 osteotomy was determined by MDT consensus, proceeding once metabolic stability (serum calcium >2.0 mmol/L; reference: 2.11–2.52 mmol/L for 48 hours) was achieved and considering the significant travel burden for the patient from a remote area. So, bilateral distal femoral lateral closing-wedge osteotomies (left: 26°, right: 18°) were performed on post-PTX day 7, with plate fixation. Postoperative management included continued calcium, magnesium, and vitamin D supplementation.

#### Follow-up

**Figure 3:**
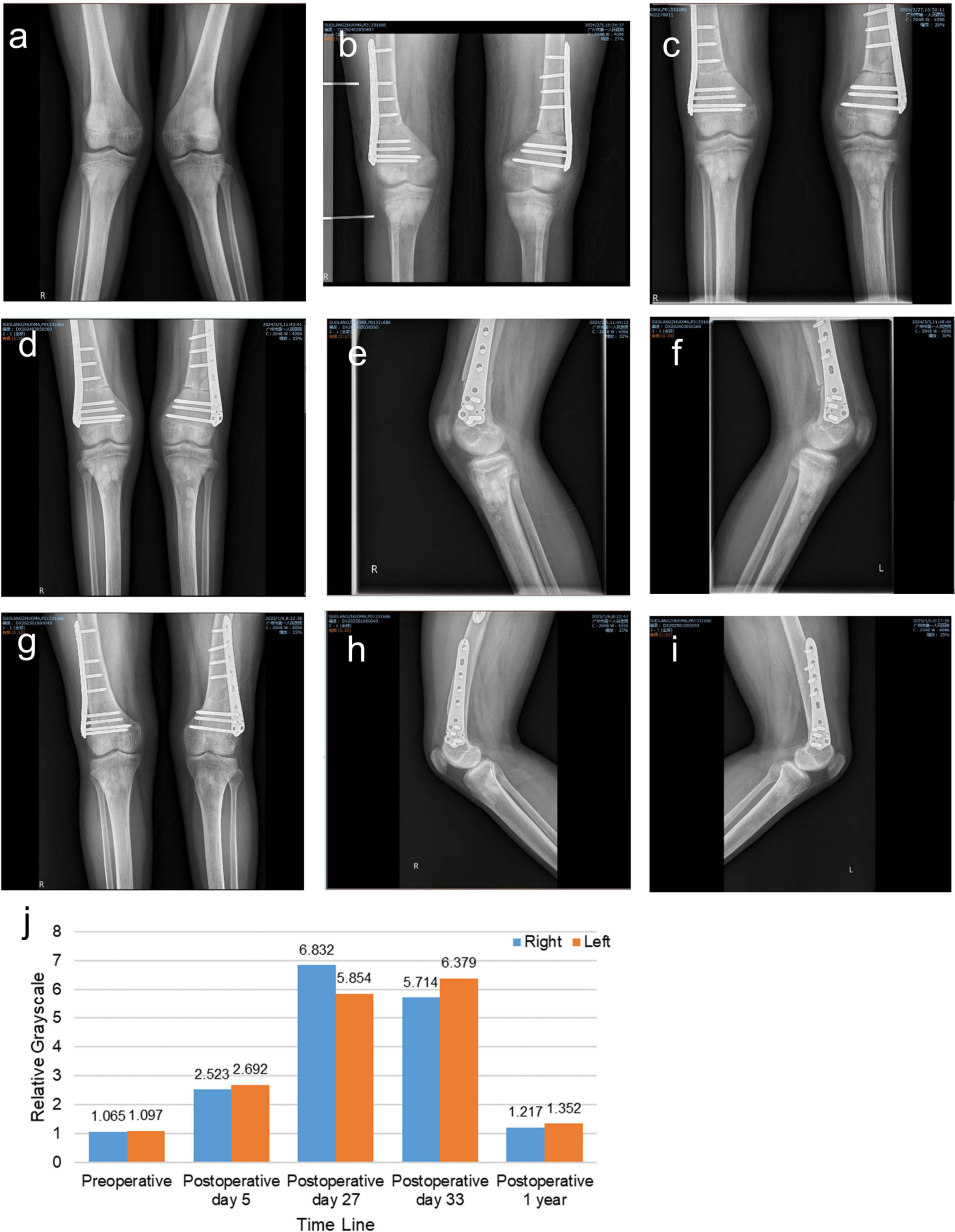
X-ray evaluation of transient osteosclerosis during the follow-up. **(a)** Preoperative radiograph showing normal bone density. **(b)** Diffuse periarticular hyperdensity (tibial metaphysis) on postoperative day 5. **(c)** Multifocal osteosclerotic regions (tibial metaphysis) emerged on postoperative day 27. **(d–f)** Stable lesions on postoperative day 33. **(g–i)** Near-complete resolution of hyperdensity at 1 year, with no pathological changes. **(j)** Quantitative analysis of transient osteosclerosis.​​ The analysis was performed using ImageJ software (National Institutes of Health, USA). Briefly, radiographs were imported, converted to 8-bit grayscale images, and two rectangular regions of interest (ROIs) of equal size were defined: one encompassing the proximal 10 cm of the tibia (including the metaphyseal sclerotic regions) and another covering the subsequent 10 cm of the tibial diaphysis distally. The relative grayscale value was calculated by dividing the mean pixel density of the proximal ROI by that of the distal ROI. The initial elevation and subsequent normalization of the relative grayscale value objectively demonstrate the appearance and resolution of the hyperdense sclerotic regions.

The patient resumed partial weight-bearing with crutches at 1 month postoperatively and achieved independent ambulation by 6 months. Radiographs at 1 year confirmed complete bony union at all osteotomy sites, with restored lower limb alignment (**Figures 2i, j**). Notably, multifocal osteosclerotic regions were observed around the knees on postoperative day 27 (**Figures 3a–c**). These findings gradually diminished in density contrast and disappeared completely by 1 year (**Figures 3d–i**), coinciding with full skeletal healing. Hardware removal was performed uneventfully at 1 year, with no recurrence of deformities. To objectively confirm the transient nature of the observed osteosclerosis, a semi-quantitative analysis of relative grayscale values was performed on serial radiographs using ImageJ software (National Institutes of Health, USA), which demonstrated an initial elevation followed by normalization in the proximal tibial metaphysis, correlating with the radiographic resolution of the lesions (**Figure 3j**).

To evaluate the possibility of familial hyperparathyroidism, the patient’s first-degree relatives (both parents and a younger sister) underwent serum calcium and parathyroid hormone (PTH) screening at a local hospital via remote medical coordination. The results for all three individuals were within normal ranges (Father: calcium 2.38 mmol/L (reference: 2.11–2.52 mmol/L), PTH 32 pg/mL (reference: 15–65 pg/mL); Mother: calcium 2.41 mmol/L, PTH 28 pg/mL; Sister: calcium 2.45 mmol/L, PTH 41 pg/mL). Furthermore, none of the relatives reported any symptoms suggestive of hypercalcemia or related endocrine disorders. Based on this normal biochemical screening and absence of clinical manifestations, an inherited form of hyperparathyroidism was considered unlikely in this kindred.

## Discussion

### Diagnostic pitfalls in atypical presentations​

The initial misdiagnosis of this case as a developmental deformity underscores the diagnostic challenges of PHPT in resource-limited settings. Two factors primarily contributed to this oversight: first, the absence of classic metabolic symptoms and only mild hypercalcemia at admission failed to prompt an endocrine workup; second, the bilateral coxa vara and genu valgus closely resembled those of skeletal disorders like nutritional rickets on radiographs, diverting diagnostic attention away from metabolic bone disease. ​Crucially, however, the pathophysiological mechanisms underlying these similar deformities are fundamentally distinct.​​ Rickets arises from defective bone mineralization, primarily due to vitamin D deficiency or abnormal metabolism, leading to accumulation of unmineralized osteoid and mechanically weakened growth plates (11). In contrast, the skeletal manifestations in PHPT are driven by excessive PTH secretion, promoting uncontrolled osteoclastic bone resorption and high bone turnover (12). The pathogenesis of such deformities in adolescents may involve PTH-mediated disruption of growth plate dynamics during pubertal growth spurts (7). Thus, the key biochemical differentiation lies in the presence of hypercalcemia and significantly elevated PTH levels in PHPT, as exemplified in our patient, which are absent in classic vitamin D-deficient rickets (11, 13). PHPT is exceptionally rare in children, and the development of genu valgum/varum requires active growth plates, making this complication exceedingly uncommon. Nevertheless, this case highlights that endocrine screening must be included in the differential diagnosis of limb deformities to avoid diagnostic delays.

The definitive diagnosis relied on histopathological confirmation. The parathyroid origin of the resected lesion was supported by strong nuclear immunoreactivity for GATA3, a highly sensitive and specific immunohistochemical marker for parathyroid tissue (14). The notably elevated Ki-67 proliferation index (~10%), while exceeding the typical range for sporadic parathyroid adenomas (15, 16), can be interpreted in the context of the lesion’s hyperplastic histology (nodular hyperplasia) and the patient’s young age. Elevated Ki-67 indices have been documented in benign parathyroid lesions, particularly in younger individuals, and may correlate with a more active proliferative state in symptomatic, early-onset disease (17, 18). This finding underscores the histological heterogeneity within hyperparathyroid pathologies and should not be solely equated with malignant potential in such a clinical setting.

It is critical to recognize that in young-onset PHPT, even when a parathyroid adenoma is identified, the possibility of underlying genetic syndromes, particularly multiple endocrine neoplasia type 1 (MEN1), must be actively considered. MEN1 is characterized by multiglandular parathyroid involvement, often presenting as hyperplasia, alongside tumors of the pancreas and pituitary gland (19, 20). Therefore, a comprehensive evaluation in such cases should extend beyond confirming the adenoma to systematically screen for hyperplasia, carcinoma, and features suggestive of MEN syndromes. In the context of familial hyperparathyroidism, genetic testing (e.g., for MEN1, CDC73 mutations) is indispensable (21, 22), not only for confirming the diagnosis but also for guiding long-term surveillance of the patient and at-risk family members for associated neoplasms.

### Surgical strategy for PHPT with severe deformities

The 2022 ASBMR guidelines advocate PTX for all patients diagnosed with PHPT who have no contraindications, including those with asymptomatic PHPT (6). We emphasize that intraoperative dynamic PTH monitoring—though not explicitly mandated in current guidelines—significantly enhances surgical success (23, 24). A >50% PTH reduction within 10–20 minutes post-excision predicts curative resection, reducing recurrent/persistent disease and reoperation rates (24). While rapid PTH and calcium decline are expected post-PTX, severe skeletal involvement predisposes to prolonged hypocalcemia (25). As exemplified by this case, daily monitoring of calcium, phosphate, and PTH during the acute postoperative phase is crucial for guiding the supplementation of calcium and active vitamin D (calcitriol). The supplementation regimen must be customized according to individual risk factors, such as the severity of baseline bone disease, to prevent the occurrence of refractory hypocalcemia (9, 26). Once calcium homeostasis is stabilized, it is advisable to gradually taper off the supplements. Subsequently, long-term surveillance is necessary, which involves annual to biannual evaluations of calcium, phosphate, PTH, renal function, urinary calcium, bone turnover markers, and bone density. This comprehensive strategy enables the early detection of recurrent or persistent hyperparathyroidism, secondary causes of bone loss, or complications like nephrolithiasis (27, 28).

The surgical correction of skeletal deformities in PHPT follows principles like those for other-etiology deformities, usually involving osteotomy and internal fixation. The decision to stage the bilateral proximal and distal femoral osteotomies in this case was due to surgical complexity, such as the wide-ranging multilevel osteotomies (bilateral proximal and distal femoral osteotomy), long operation time, and expected high blood loss. However, the optimal time for orthopedic intervention—either doing corrective surgery along with PTX or delaying until bone turnover normalizes after PTX—is still uncertain (7, 29). In this case, orthopedic correction started right after hypocalcemia was stabilized with intravenous calcium post-PTX. Remarkably, all osteotomy sites achieved radiographic union within 1 year. This shows that successful bone healing may not rely solely on normal bone metabolism. Instead, it depends on meticulous peri-operative management, including aggressive calcium and vitamin D supplementation, weight-bearing limits, and close biochemical monitoring. These results challenge the common view of delaying orthopedic surgery until metabolic parameters are stable and show the possibility of early intervention in certain patients (30, 31). However, more studies are needed to set criteria for safe surgical timing, considering both metabolic and procedural risks.

### Pathophysiological considerations for postoperative biochemical dynamics and management

The marked elevation of serum calcium and PTH following the initial orthopedic surgery represents a notable and instructive phenomenon. We postulate that this surge likely resulted from the convergence of two key mechanisms. First, the concept of the ‘hungry bone’ state and its postoperative reversal played a central role (32). Preoperatively, chronic PTH excess had led to significant skeletal demineralization. The first-stage osteotomy and subsequent immobilization likely caused an acute shift in bone turnover: while disuse may have transiently moderated osteoclastic activity, the surgical trauma and initiation of bone repair at the osteotomy sites created a sudden, high demand for calcium and phosphate for remineralization (33, 34). This rapid influx of minerals into the skeleton could have lowered serum ionized calcium, providing a potent stimulus to any residual abnormal parathyroid tissue to secrete more PTH in an attempt to defend serum calcium levels. Second, the systemic stress and inflammatory response inherent to major surgery cannot be overlooked. Inflammatory cytokines released during the postoperative period can indirectly influence calcium homeostasis and may potentiate PTH secretion (35). This interplay between skeletal mineral demand and surgical stress provides a plausible explanation for the observed acute biochemical exacerbation.

Correspondingly, our management strategies for both the ensuing hypercalcemia and the subsequent post-parathyroidectomy hypocalcemia were tailored to these acute pathophysiological shifts.​ For the postoperative hypercalcemia, while bisphosphonates and calcimimetics are established first-line agents (36–38), the selection of calcitonin was context-dependent. The priority was rapid calcium reduction while arranging definitive surgery. Calcitonin, with its rapid onset of action, served as an effective short-term bridge (39, 40), chosen over bisphosphonates due to concerns of potential nephrotoxicity in the acute postoperative setting and the unavailability of calcimimetics.

Following curative parathyroidectomy, the profound hypocalcemia reflected the culmination of the “hungry bone” state. The therapeutic regimen of high-dose intravenous calcium gluconate concurrent with a relatively low initial dose of oral calcitriol was a deliberate, physiology-driven approach. High-dose IV calcium was essential for the immediate, titratable correction of life-threatening hypocalcemia. The cautious initiation of calcitriol aimed to gradually stimulate intestinal calcium absorption—a slower, more physiological process—to sustainably meet the skeleton’s intense mineral avidity while minimizing the risks of overcorrection, hypercalciuria, and nephrolithiasis. This staged strategy prioritized immediate safety with IV calcium while using calcitriol to carefully rebuild long-term calcium homeostasis, with both agents meticulously titrated against frequent biochemical monitoring.

### Transient osteosclerosis as a novel postoperative phenomenon

Interestingly, imaging revealed transient osteosclerotic regions around the knees during the follow-up in this case. This finding, while uncommon, aligns with the broader spectrum of PHPT-associated skeletal changes, where osteosclerosis has been historically described, most frequently in the skull and vertebrae, and rarely in the metaphyses of long bones in children (41). The pathophysiology in such cases is thought to involve an imbalance between bone formation and resorption under chronic PTH excess (42). However, our case presents distinct and novel features. Unlike the chronic, often axial (spinal/pelvic) sclerosis reported in long-standing adult PHPT or the metaphyseal sclerosis occasionally seen in pediatric cases preoperatively, the osteosclerotic lesions in our adolescent patient were multifocal, predominantly metaphyseal (peri-knee), and emerged in the postoperative period. We propose that these radiographic changes represent a transient state of hyper-mineralization, driven by a sudden postoperative shift in bone balance. The rapid drop in PTH after surgery sharply reduces bone breakdown by osteoclasts. At the same time, aggressive calcium and vitamin D supplementation strongly promotes mineral deposition into the skeleton. Areas with naturally high turnover, like the metaphyses around the knees, likely become the primary sites for this mineral influx (12, 43). This creates a temporary imbalance where bone formation outpaces resorption, leading to the dense patches seen on X-rays. The close timing of these changes with calcium treatment, followed by their complete resolution, indicates a self-limiting, reparative process rather than a new disease. Although iatrogenic calcification due to surgical trauma cannot be entirely excluded, the bilateral symmetry and lack of correlation with osteotomy sites argue against this mechanism. Additionally, the transient osteosclerosis observed in our case differs fundamentally from sclerotic bone changes seen in other endocrine disorders. In pseudohypoparathyroidism, sclerosis typically occurs in the setting of PTH resistance, characterized by hypocalcemia and elevated PTH levels, and often presents as progressive subcutaneous ossification or basal ganglia calcification rather than metaphyseal hypermineralization (44). In renal osteodystrophy, a mixed pattern of osteosclerosis and osteolysis is common, resulting from a combination of secondary hyperparathyroidism and altered vitamin D metabolism in chronic kidney disease (45). In contrast, our patient’s transient, self-resolving osteosclerosis emerged in the context of a rapid biochemical cure of PHPT, supporting a distinct mechanism related to postoperative mineral redistribution.

There are several limitations in this study. First, the absence of pre- and postoperative bone mineral density (BMD) assessment precludes a quantitative evaluation of skeletal recovery and longitudinal monitoring of bone metabolism following parathyroidectomy. Second, the transient osteosclerotic lesions observed around the knees lacked histopathological confirmation, leaving their precise etiology—such as localized mineralization versus reactive changes—speculative. Although their spontaneous resolution suggests a benign process, the lack of biopsy or advanced imaging limits deeper mechanistic insights. Third, potential ethnic-specific characteristics of primary hyperparathyroidism in the Tibetan population and the influence of high-altitude geographical factors were not explored. Fourth, the biochemical workup was constrained by the lack of timely vitamin D level assessment and the unavailability of specific bone turnover markers (e.g., osteocalcin, C-telopeptide of type I collagen), which restricted further subclassification of PHPT and a detailed analysis of bone remodeling dynamics. Finally, germline genetic testing for hereditary syndromes (e.g., MEN1, CDC73), which was strongly indicated by the patient’s young age and histologic findings, could not be performed. This was primarily due to the compounded challenges of the patient’s remote geographical origin in rural Tibet, where access to specialized genetic services is extremely limited, and despite comprehensive counseling and repeated recommendations, informed consent for testing could not be obtained from the family. As a single-case report, our observations require validation in larger cohorts to establish generalizability.

## Conclusions

This report elucidates the diagnostic and therapeutic complexities of severe, pediatric-onset PHPT presenting with skeletal deformities. It underscores the critical importance of including metabolic bone disease in the differential diagnosis of atypical limb deformities to prevent diagnostic delay. The case demonstrates that successful orthopedic correction can be achieved before the complete normalization of bone metabolism, provided it is supported by rigorous perioperative management of calcium and vitamin D homeostasis. Finally, we identify and characterize transient postoperative osteosclerosis​ as a previously unreported radiographic finding, proposing it as a unique, reparative skeletal response to the abrupt correction of severe hyperparathyroidism. This phenomenon enriches the understanding of postoperative bone remodeling and warrants further investigation.

## Data Availability

The original contributions presented in the study are included in the article/**Supplementary Material**. Further inquiries can be directed to the corresponding authors.
