# Supplementary material for: Case Report: Developmental-like skeletal deformities and transient osteosclerosis as rare presentations of primary hyperparathyroidism
Source: Front Endocrinol (Lausanne). 2026 Feb 13;17:1672914. doi: 10.3389/fendo.2026.1672914 (PMC12945759; doi:10.3389/fendo.2026.1672914)
Supplement: Supplementary file 1 [file SupplementaryFile1.docx]

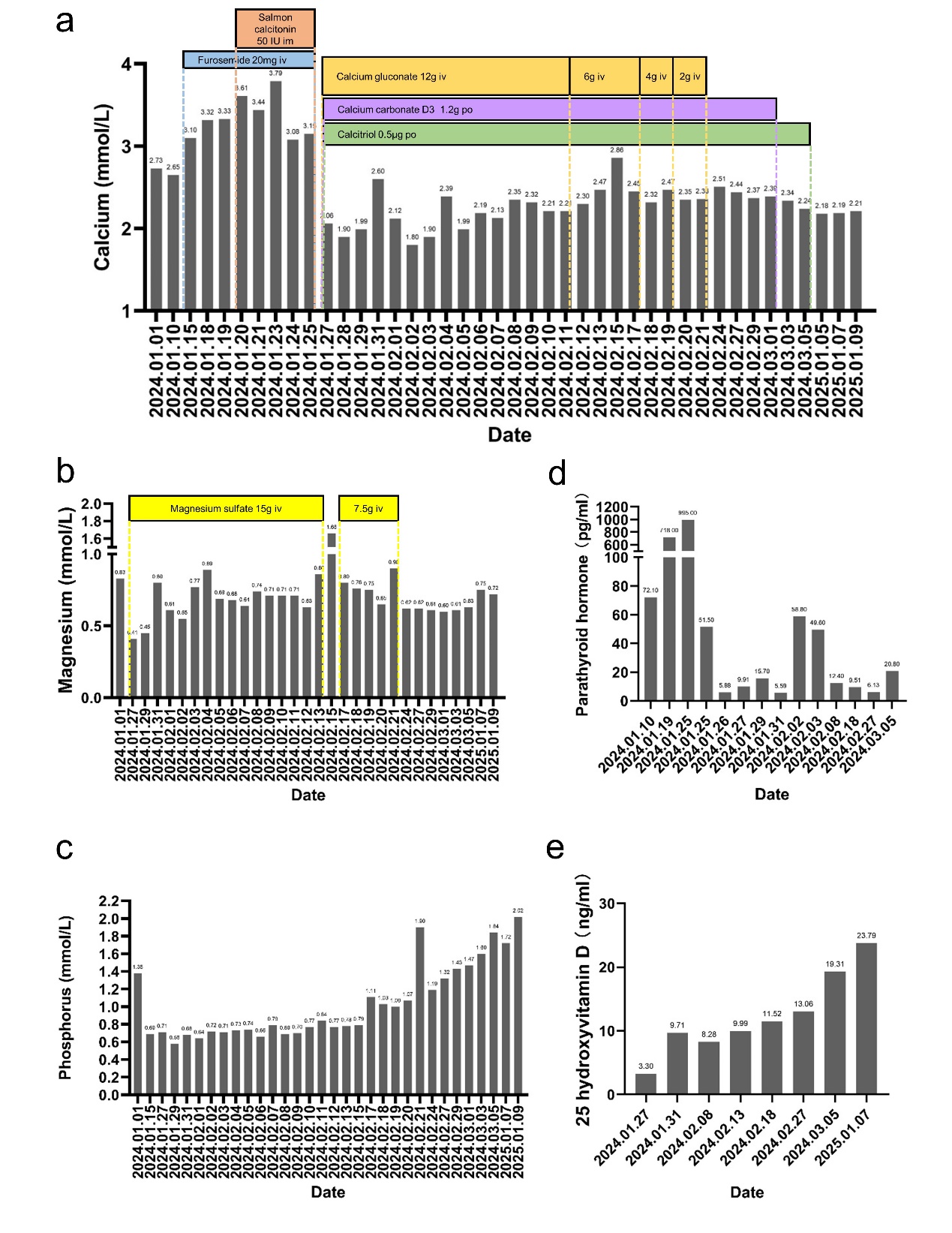


**Supplementary Figure 1. Dynamic biochemical monitoring and therapeutic regimen.** (a) Calcium management: Hypercalcemia was detected on January 15, 2024 (the 5th day after the first-stage osteotomy). Furosemide was administered intravenously at a dose of 20 mg per day until a parathyroidectomy (PTX) was performed on January 25, 2024. Salmon calcitonin was added for intramuscular injection at a dose of 50 IU per day starting from January 20, 2024, until the PTX on January 25, 2024. Hypocalcemia occurred on January 27, 2024 (the 2nd day after the PTX. Intravenous calcium gluconate was administered at a dose of 12 g per day. The dose was adjusted to 6 g per day on February 12, 2024, and then to 2 g per day on February 20, 2024, before the drug was discontinued on February 21, 2024. In addition, oral calcium carbonate was administered at a dose of 1.2 g per day starting from January 27, 2024, until it was discontinued on March 1, 2024. (b) Magnesium management: Hypomagnesemia occurred on January 27, 2024 (the 2nd day after the PTX. Intravenous magnesium sulfate was administered at a dose of 15 g per day. The drug was discontinued due to elevated serum magnesium levels on February 13, 2024. Intravenous magnesium sulfate was restarted at a dose of 7.5 g per day on February 17, 2024, due to recurrent hypomagnesemia and was discontinued on February 21, 2024, when the serum magnesium level returned to normal. (c-e) Dynamic monitoring results of serum phosphorus, parathyroid hormone (PTH), and 25-hydroxyvitamin D during the patient's hospitalization. Reference: calcium: 2.11–2.52 mmol/L; Magensium: 0.7–1.1 mmol/L ; Phosphorus: 0.85–1.51 mmol/L; parathyroid hormone: 15–65 pg/mL; 25 hydroxyvitamin D: 20-100 ng/mL
